# Netrin-1 Is an Important Mediator in Microglia Migration

**DOI:** 10.3390/ijms25137079

**Published:** 2024-06-27

**Authors:** Hua-Li Yu, Xiu Liu, Yue Yin, Xiao-Nuo Liu, Yu-Yao Feng, Muhammad Mateen Tahir, Xin-Zhi Miao, Xiao-Xiao He, Zi-Xuan He, Xiao-Juan Zhu

**Affiliations:** Key Laboratory of Molecular Epigenetics, Ministry of Education and Institute of Cytology and Genetics, Northeast Normal University, Changchun 130024, China; yuhl178@nenu.edu.cn (H.-L.Y.); liux902@nenu.edu.cn (X.L.); yiny340@nenu.edu.cn (Y.Y.); liuxn757@nenu.edu.cn (X.-N.L.); fengyy553@nenu.edu.cn (Y.-Y.F.); mat540@nenu.edu.cn (M.M.T.); miaoxz745@nenu.edu.cn (X.-Z.M.); hexx100@nenu.edu.cn (X.-X.H.); hezx234@nenu.edu.cn (Z.-X.H.)

**Keywords:** cerebral cortex, microglia migration, Netrin-1, Integrin α6β1, GSK3β

## Abstract

Microglia migrate to the cerebral cortex during early embryonic stages. However, the precise mechanisms underlying microglia migration remain incompletely understood. As an extracellular matrix protein, Netrin-1 is involved in modulating the motility of diverse cells. In this paper, we found that Netrin-1 promoted microglial BV2 cell migration in vitro. Mechanism studies indicated that the activation of GSK3β activity contributed to Netrin-1–mediated microglia migration. Furthermore, Integrin α6/β1 might be the relevant receptor. Single-cell data analysis revealed the higher expression of Integrin α6 subunit and β1 subunit in microglia in comparison with classical receptors, including Dcc, Neo1, Unc5a, Unc5b, Unc5c, Unc5d, and Dscam. Microscale thermophoresis (MST) measurement confirmed the high binding affinity between Integrin α6/β1 and Netrin-1. Importantly, activation of Integrin α6/β1 with IKVAV peptides mirrored the microglia migration and GSK3 activation induced by Netrin-1. Finally, conditional knockout (CKO) of Netrin-1 in radial glial cells and their progeny led to a reduction in microglia population in the cerebral cortex at early developmental stages. Together, our findings highlight the role of Netrin-1 in microglia migration and underscore its therapeutic potential in microglia-related brain diseases.

## 1. Introduction

Microglia, the resident macrophages of the central nervous system (CNS), originate from yolk sac hematopoietic progenitors and migrate to the brain during early embryonic stages [1]. Traditionally recognized for their roles in immune responses to infection or injury, recent research has illuminated their unique contributions to brain development. These include the control of neural progenitor cell numbers [2], promotion of neurogenesis and oligodendrogenesis [3], orchestration of neural circuit formation [4,5], and elimination of excess synapses [6,7]. Importantly, evidence from human studies implicates aberrant microglia development and function in neurodevelopmental disorders such as autism and schizophrenia [8,9].

Microglia colonization of the cerebral cortex occurs between embryonic day 10.5 (E10.5) and E17.5 in mice. This process is regulated by various signals from CNS cells. Programmed neuronal apoptosis is one contributing factor to microglia colonization [10], while chemokines secreted by neural progenitors and developing neurons, such as CXCL12, CSF1, and IL-34, facilitate microglia migration into the cerebral cortex [11,12]. Despite these advances, the precise mechanisms underlying microglia migration into the cerebral cortex remain incompletely understood, leaving room for further exploration of novel regulators.

Netrin-1, a secreted protein highly expressed in the nervous system, is traditionally known for its role as a guidance cue in regulating neuronal migration and axon projection independent of different receptors, such as DCC and UNC5 [13,14,15]. Interestingly, Netrin-1 has also been implicated in modulating immune cell motility, inhibiting macrophage emigration from atherosclerotic plaques [15], and limiting neutrophil recruitment in acute colitis models [16]. Given that microglia are immune cells within the CNS, we sought to investigate the potential involvement of Netrin-1 in microglia migration.

Here, our study revealed that conditional knockout (CKO) of Netrin-1 in radial glial cells and their progeny led to a reduction in microglia population in the cerebral cortex, with levels returning to normal by postnatal day 14 (P14). In vitro experiments utilizing the murine microglial BV2 cell line unveiled that Netrin-1 application increased BV2 migration and GSK3β activity. Further investigation demonstrated that suppression of GSK3β activity hindered Netrin-1-induced BV2 migration. Single-cell data analysis indicated that Integrin α6/β1 served as the relevant receptor, as Integrin α6/β1 was capable of binding to Netrin-1, and activation of Integrin α6/β1 with IKVAV peptides mirrored the migration and GSK3β activation induced by Netrin-1. Taken together, our findings highlight the role of Netrin-1 in promoting microglia migration, both in vitro and in vivo.

## 2. Results

### 2.1. Netrin-1 Promotes BV2 Elongation and Migration

**Figure 1:**
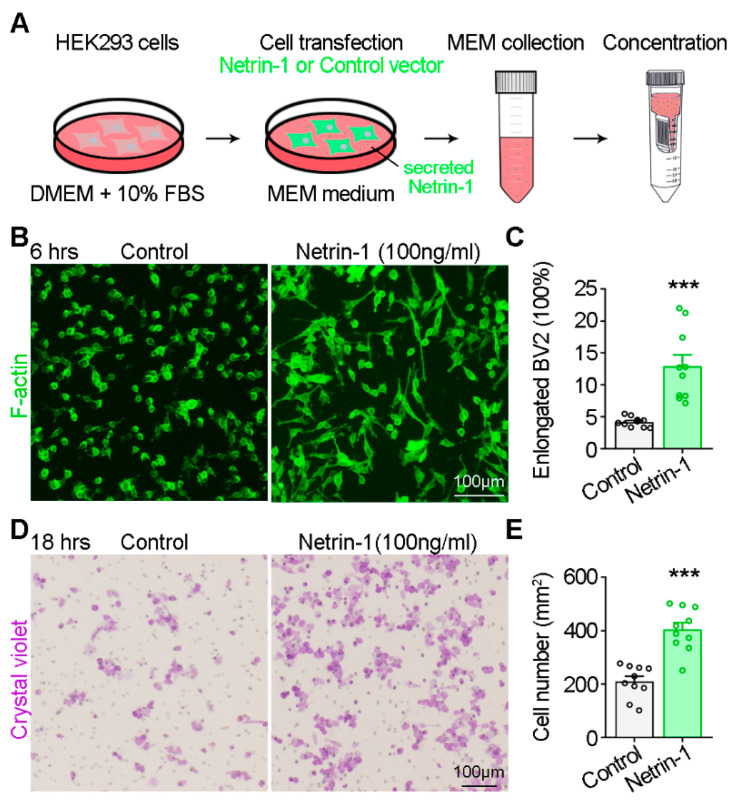
Promotional effect of Netrin-1 on BV2 cell migration. (**A**) Model of Netrin-1 conditioned medium preparation. (**B**) Representative images showing BV2 cell morphology in response to Netrin-1 stimulation (6 h). (**C**) Quantification of the percentage of elongated BV2 cells. Student’s t test, *p* = 0.0001. (**D**) Trans-well assay showing the migration of BV2 cells in application of Netrin-1 (18 h). (**E**) Quantification of the number of migrated BV2 cells. Student’s *t* test, *p* < 0.0001. The data are from at least three independent experiments. *** *p* < 0.001.

To investigate the impact of Netrin-1 on microglia migration, we initially assessed its effect on BV2 microglial cells in vitro. Netrin-1 conditioned medium was prepared as described previously [17] (Figure 1A). Following a 6h incubation with Netrin-1 conditioned medium, BV2 cells transitioned from a rounded morphology to a spindle-like shape, displaying one or two elongated processes (Figure 1B). We speculated that this polarized morphology might facilitate the directed migration of microglia. Further analysis using the trans-well migration assay demonstrated that Netrin-1 enhanced BV2 migration (Figure 1C).

### 2.2. Netrin-1 Increases GSK3β Activity by Suppressing GSK3β Phosphorylation at Serine 9

**Figure 2:**
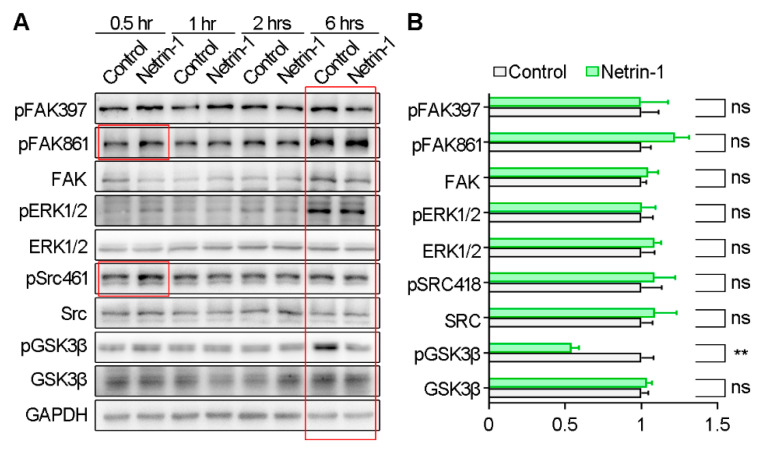
The increase of GSK3β activity by Netrin-1. (**A**) Western blotting showing the activity of kinases as indicated after Netrin-1 stimulation. (**B**) Quantification of the phosphorylated levels of indicated kinases. Student’s *t* test, *p* < 0.01 for pGSK3β. Data are presented as the mean ± SEM. The data are from at least three independent experiments. ns, no significant difference; ** *p* < 0.01.

Given the multifaceted regulation of cell migration by various signaling pathways, we investigated the activation of downstream pathways following Netrin-1 stimulation using Western blotting (Figure 2A). We observed an increase in the phosphorylation of FAK-Y861 and Src-T416 after 30 min of Netrin-1 incubation, confirming the efficacy of Netrin-1 conditioned medium [18]. Particularly noteworthy was the reduction in GSK3β phosphorylation at Serine 9 (pGSK3β-Ser9) induced by 6 h incubation of Netrin-1 (Figure 2B). As Ser9 phosphorylation inhibited GSK3β kinase activity, it indicates heightened GSK3β kinase activity in response to Netrin-1.

### 2.3. Netrin-1 Regulates BV2 Elongation and Migration through Activating GSK3β Activity

**Figure 3:**
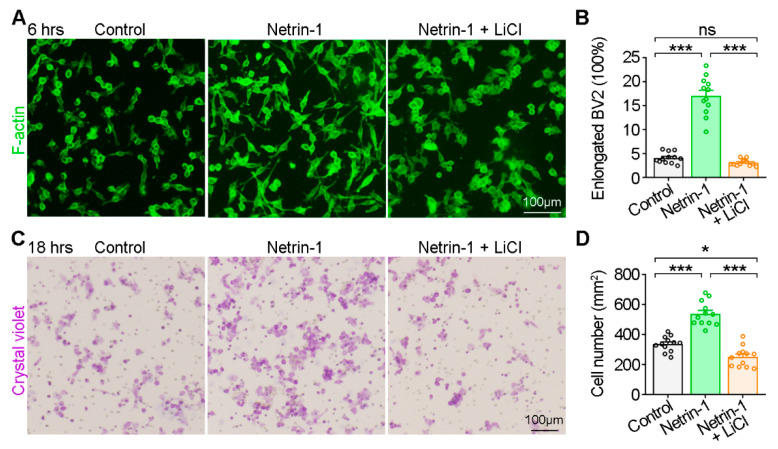
Suppression of Netrin-1 mediated BV2 migration by GSK3β inhibitor. (**A**) Representative images showing BV2 cell morphology after Netrin-1 stimulation with or without LiCl (6 h). (**B**) Quantification of the percentage of elongated BV2 cells. One-way ANOVA, for Control and Netrin-1 groups, *p* < 0.0001; for Control and Netrin-1 + LiCl groups, *p* = 0.3314; for Netrin-1 and Netrin-1 + LiCl groups, *p* < 0.0001. (**C**) Trans-well assay showing the migration of BV2 cells after Netrin-1 stimulation with or without LiCl (18 h). (**D**) Quantification of the number of migrated BV2 cells. One-way ANOVA, for Control and Netrin-1 groups, *p* < 0.0001; for Control and Netrin-1 + LiCl groups, *p* = 0.0102; for Netrin-1 and Netrin-1 + LiCl groups, *p* < 0.0001. The data are from at least three independent experiments. ns, no significant difference; * *p* < 0.05; *** *p* < 0.001.

To corroborate that the observed BV2 cell phenotype induced by Netrin-1 was mediated via GSK3β activation, BV2 cells were treated with Netrin-1 conditioned medium in the presence of LiCl, a GSKβ inhibitor. Our results revealed that LiCl treatment abrogated the elongation of BV2 cells triggered by Netrin-1 application (Figure 3A,B). Consistently, the trans-well migration assay revealed that LiCl treatment also suppressed the migration of BV2 cells triggered by Netrin-1 application (Figure 3C,D). These data underscore the dependency of Netrin-1-induced BV2 elongation and migration on GSK3β activation.

### 2.4. Integrin α6/β1 Is Highly Expressed in Microglia and Interacts with Netrin-1

**Figure 4:**
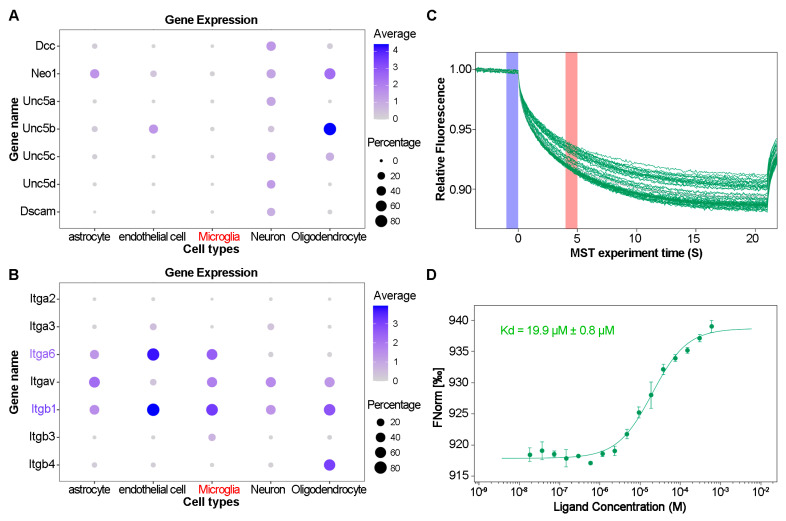
The high expression of Integrin α6β1 in microglia as well as the interaction between Integrin α6β1 and Netrin-1. (**A**) Single-cell data from Tabula Muris showing the expression level of Netrin-1 traditional receptors, involving Dcc, Neo1, Unc5a, Unc5b, Unc5c, Unc5d, and Dscam in mouse microglia. (**B**) Single-cell data from Tabula Muris showing the expression level of Integrin subunits associated with Netrin-1. (**C**) Multiple MST traces for different mixture ratios of Integrin α6β1 and Netrin-1. (**D**) Dose–response analysis of the MST traces for determination of the steady-state affinity of the Integrin α6β1–Netrin-1 interaction.

To elucidate the receptors mediating GSK3β activation, we examined the expression of Netrin-1 receptors in microglia. Utilizing single-cell data (Tabula Muris) [19], we observed minimal expression of classical receptors such as Dcc, Neo1, Unc5a, Unc5b, Unc5c, Unc5d, and Dscam in microglia (Figure 4A). Since some Integrins are considered receptors of Netrin-1, we also analyzed the expression of Integrin subunits associated with Netrin-1, which included Integrin α2, α3, α6, αν, β1, β3, and β4 [20,21,22,23]. Of note, Integrin β1 and Integrin α6 exhibited high expression levels (Figure 4B), suggesting a potential role for Integrin α6β1 as the dependent receptor. To confirm the interaction between Integrin α6β1 and Netrin-1, we performed MST assay using RED dye-labeled Integrin α6β1 proteins and unlabeled Netrin-1 proteins. It was indicated that Netrin-1 had a high binding affinity with Integrin α6β1 (Figure 4C,D). The dissociation constant was 19.9 μM ± 0.8 μM.

### 2.5. Activating Integrin α6/β1 Mimics Netrin-1-Induced GSK3β Activity as well as BV2 Elongation and Migration

**Figure 5:**
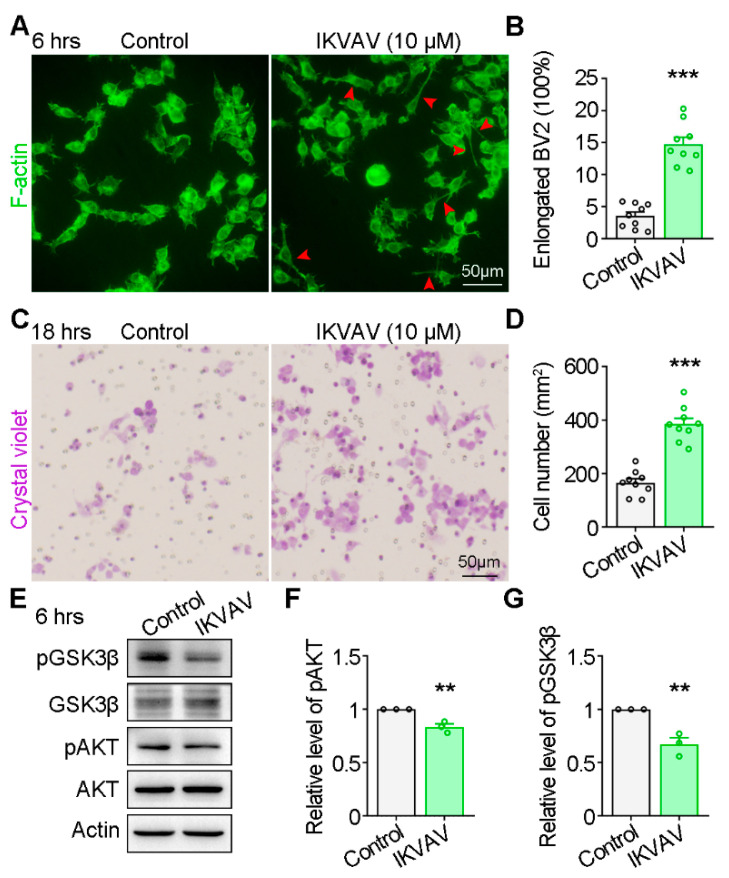
IKVAV mimicking the effects of Netrin-1 on BV2 migration and GSK3β activation. (**A**) Representative images showing BV2 cell morphology in response to IKVAV stimulation (6 h). (**B**) Quantification of the percentage of elongated BV2 cells. Student’s *t* test, *p* < 0.0001. (**C**) Trans-well assay showing the migration of BV2 cells in application of IKVAV (18 h). (**D**) Quantification of the number of migrated BV2 cells. Student’s *t* test, *p* < 0.0001. (**E**) Western blotting showing the activity of AKT and GSK3 after IKVAV stimulation. (**F**) Quantification of the phosphorylated levels of AKT. Student’s *t* test, *p* = 0.0052. (**G**) Quantification of the phosphorylated levels of GSK3β. Student’s *t* test, *p* = 0.0059. Data are presented as the mean ± SEM. The data are from at least three independent experiments. ** *p* < 0.01; *** *p* < 0.001.

To examine the involvement of Integrin α6/β1 in GSK3β activity, BV2 cell elongation, and migration, we stimulated BV2 cells with isoleucine-lysine-valine-alanine-valine (IKVAV) peptides, known to activate Integrin α6/β1 [24]. Following a 6 h treatment with IKVAV, the percentage of spindle-like BV2 cells increased (Figure 5A,B), along with enhanced BV2 cell migration (Figure 5C,D). Moreover, IKVAV treatment led to a reduction in GSK3β phosphorylation at Serine 9 and AKT phosphorylation, an upstream kinase of GSK3β (Figure 5E–G). These findings demonstrate that activating Integrin α6/β1 promotes BV2 elongation and migration as well as GSK3β activation, mirroring the effects induced by Netrin-1.

### 2.6. Netrin-1 Is Highly Expressed in the Developing Cerebral Cortex

**Figure 6:**
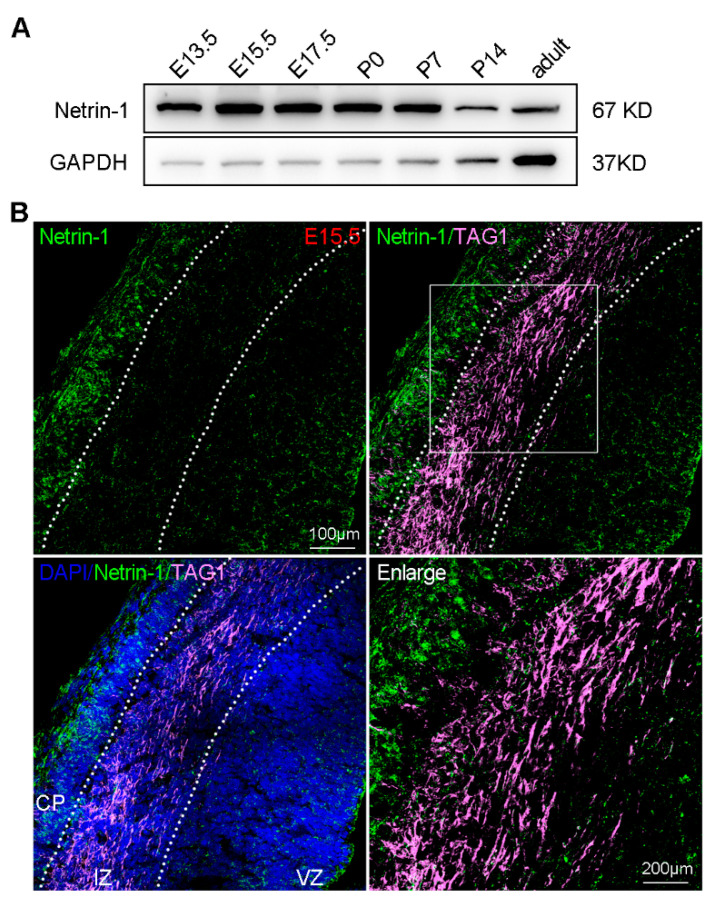
The expression and distribution of Netrin-1 in developing cerebral cortex. (**A**) Western blotting showing the expression level of Netrin-1 at indicated developmental stages. (**B**) The distribution of extracellular Netrin-1 in E15.5 brain sections.

To further explore the role of Netrin-1 in microglia in vivo, we assessed its expression and distribution in the developing cerebral cortex. Western blotting revealed high expression levels of Netrin-1 in the cerebral cortex from E13.5 to P7 and low expression levels of Netrin-1 in the cerebral cortex from P14 to adult (Figure 6A), suggesting a role for Netrin-1 in early brain development. Additionally, we analyzed the distribution of Netrin-1 at E15.5. We used DAPI and anti-TAG1 antibody to identify the cortical plate (CP), intermediate zone (IZ), and ventricular zone (VZ). Extracellular Netrin-1 was observed to accumulate in the CP and VZ, regions adjacent to the meninges and ventricles, through which microglia enter the cerebral cortex (Figure 6B). The expression and distribution pattens of Netrin-1 suggest a possible role of Netrin-1 as a local cue in microglia migration at early developmental stages of the cerebral cortex.

### 2.7. Netrin-1 Knockout Suppresses Microglia Migration into the Cerebral Cortex during Early Brain Development

**Figure 7:**
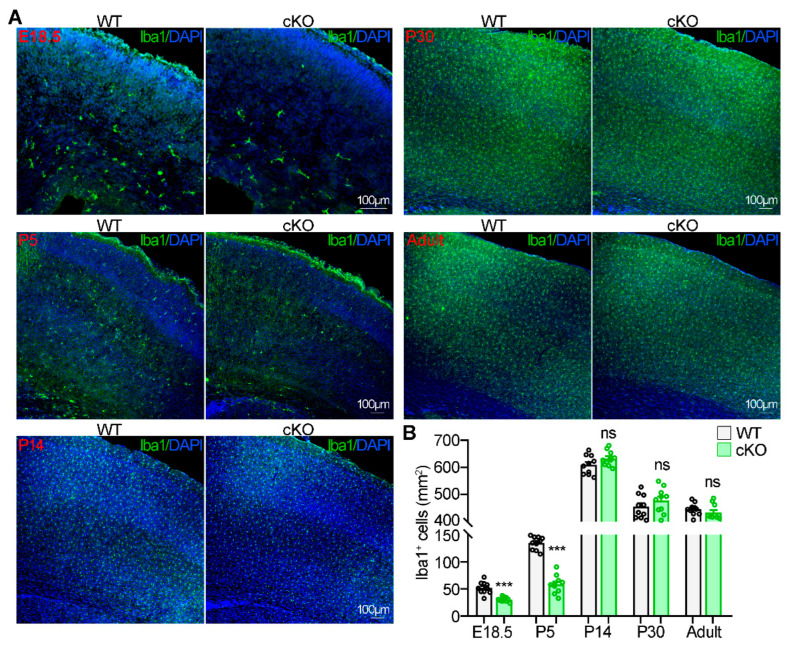
The decrease of Iba1^+^ microglia in the developing cerebral cortex of *Ntn1* cKO mice. (**A**) Immunostaining showing the number of Iba1^+^ microglia in the cortical brain at indicated developmental stages. (**B**) Quantification of the microglia numbers. For each group, 10 sections from 3 mice were used for statistical analysis. Student’s *t* test, for E18.5, *p* < 0.0001; for P5, *p* < 0.0001; for P14, *p* = 0.1159; for P30, *p* = 0.2692; for P30, *p* = 0.3057. Data are presented as the mean ± SEM. The data are from at least three independent experiments. ns, no significant difference; *** *p* < 0.001.

Subsequently, we crossed *Ntn1*^flox/flox^ mice with GFAP-Cre mice to delete *Ntn1* in radial glial cells and their progeny [14]. *Ntn1*^flox/flox^;GFAP-Cre (cKO) mice and their wild-type (WT) littermates were sacrificed at different time points to examine microglia distribution in the cerebral cortex. At E18.5 and P5, the number of microglia in the cerebral cortex was reduced in cKO mice compared to WT mice (Figure 7A,B). However, from P14 to adulthood, the number of microglia in the cerebral cortex of cKO mice was comparable to that of WT mice, suggesting that microglia in cKO mice returned to normal levels after P14 (Figure 7A,B). The microglia distribution pattern was consistent with Netrin-1 expression changes. These results demonstrate that Netrin-1 is crucial for microglia migration into the cerebral cortex during embryonic and early life stages.

## 3. Discussion

In this study, we demonstrated that Netrin-1 promotes microglia migration both in vitro and in vivo, suggesting an important role for Integrin α6/β1 as the receptor mediating Netrin-1-induced GSK3β activation, thereby facilitating microglia migration.

The expression pattern of Netrin-1 in the cerebral cortex undergoes dynamic changes, reflecting its diverse roles throughout development (Figure 6). From E13.5 to P5, Netrin-1 is highly expressed, promoting neuronal migration [13] and axon initiation [14]. Between P5 and P14, the level of Netrin-1 gradually decreases, facilitating axon branching [14] and synapse formation [25]. In adulthood, once brain development is fully completed, Netrin-1 expression remains at a very low level. Interestingly, microglia numbers exhibit a similar trend to Netrin-1 expression. During embryonic stages, microglia numbers in the cerebral cortex gradually increase, mainly due to migration through vasculature [26]. Following the completion of the blood–brain barrier after birth, microglia can no longer migrate into the nervous system; their numbers increase through proliferation, peaking at P14 [27]. This expansion phase is then followed by a decrease in cell numbers due to a reduction in proliferative capacity and an increase in apoptosis [27]. In our study, we observed that microglia in WT mice exhibited the aforementioned dynamic changes (Figure 7). In contrast, in Netrin-1 conditional knockout (cKO) mice, a reduction in microglia numbers in the cerebral cortex was observed at E18.5 and P5, with numbers returning to normal levels by P14 (Figure 7). This suggests that the reduction in microglia numbers is primarily due to impaired migration rather than proliferation. Since the increase of microglia numbers is proportional to the brain growth, it is supposed that the restoration of microglia numbers in the cerebral cortex of cKO mice to WT levels may be due to the space availability [28].

Indeed, Netrin-1 is broadly expressed in the brain, present in radial glial cells, neurons, astrocytes, oligodendrocytes, pericytes, and endothelial cells. Our study revealed that Netrin-1, particularly from radial glial cells and their neuronal and astrocytic progeny, plays a crucial role in microglia migration to the cerebral cortex. However, it is possible that Netrin-1 from other cell types, such as oligodendrocytes, also contributes to microglia distribution [29]. Further studies are required to elucidate this issue. Recently, Netrin-1 has been reported to accumulated along axon projections [30]. In the cerebral cortex, Netrin-1 is distributed along corpus callosum axons [29,31], where microglia are known to accumulate nearby [32,33]. This spatial correlation supports our view that Netrin-1 is an important mediator for microglia migration and distribution.

Integrins are transmembrane heterodimeric glycoproteins involved in cell–cell and cell–extracellular matrix adhesion, transmitting signals from the extracellular environment to the cytoskeleton. Integrin α6/β1 serves as a selective laminin receptor, which has been implicated in various cellular migrations, including those of tumor cells [34], endothelial cells [35], neural precursor cells [36], and helper T cells [37]. Single-cell sequencing data indicated high expression of Integrin α6 and β1 in microglia (Figure 4A,B), suggesting an important role of Integrin α6/β1 in microglia. Netrin-1, resembling laminin, is reported to bind to both β1 subunit and α6 subunit [22,23]. In this study, we provided the direct evidence for the interaction between Integrin α6/β1 and Netrin-1 by MST assay (Figure 4C,D). Thus, we speculated that Integrin α6/β1 might be the dependent receptor for Netrin-1-induced microglia migration. To validate the speculation, we used IKVAV, a laminin-derived peptide, to stimulate BV2 cells. It was previously reported that Integrin α6/β1 could be activated by IKVAV [24]. We then analyzed the BV2 cell morphology and BV2 cell migration in the application of IKVAV. As expected, IKVAV induced microglia elongation and migration, akin to the effect elicited by Netrin-1 conditioned medium (Figure 1 and Figure 5). It is suggested that Integrin α6/β1 is the dependent receptor for Netrin-1-induced microglia morphology changes and migration.

GSK3β, a downstream target of Integrins [38], regulates multiple cellular functions, such as cell survival, proliferation, adhesion, and migration. In its native form, GSK3β is constitutively active and becomes inactivated by phosphorylation at Ser9 [39]. Our findings revealed that Netrin-1 conditioned medium decreased pGSK3β-Ser9, indicating increased GSK3β activity (Figure 2). Inhibition of GSK3β activity by LiCl suppressed BV2 migration, underscoring the role of GSK3β activation in Netrin-1-induced BV2 elongation and migration (Figure 3). It is reported that GSK3β activation contributes to cell migration by promoting cell adhesion and regulating cytoskeleton dynamics [40]. Phosphorylation of FAK861, a downstream effector of GSK3β, was increased upon Netrin-1 stimulation, indicative of enhanced cell adhesion. Moreover, the elongation of BV2 cells suggested augmented microtubule assembly and stability, implicating GSK3β in cytoskeletal motility regulation.

The significance of Netrin-1 regulation of microglia migration is not limited to the developmental stage; it is also important for certain microglia-related diseases in adulthood. In adults, microglia have a ramified morphology, with multiple processes protruding and retracting continuously to survey brain homeostasis. Upon injury or infection, microglia become activated and migrate towards the injury or infection site, where they exhibit dual functions of brain protection through phagocytosis and brain damage via inflammation cytokine release [41]. Recent studies have explored the potential of Netrin-1 in stroke treatment, suggesting its ability to inhibit microglia activation and inflammation cytokine release in a hemorrhagic stroke rat model [42]. Our findings indicate that Netrin-1 enhances microglia migration, further highlighting its therapeutic potential in stroke.

In summary, our study unveils a role for Netrin-1 in promoting microglia migration into the developing cerebral cortex. These insights shed light on the mechanisms underlying Netrin-1-mediated microglia colonization and underscore its therapeutic significance in microglia-related brain diseases.

## 4. Materials and Method

### 4.1. Animals

All animal experiments were approved by the Institutional Animal Care and Use Committee of Northeast Normal University (NENU/IACUC, AP20210315). National Standards of the People’s Republic of China (GB/T 35892–2018), Laboratory Animal—Guideline for Ethical Review of Animal Welfare, was the guidance for our animal care protocols. *Ntn1*^flox/flox^ mice were generated as previously described [14,43]. All mice were bred on the C57BL/6 background.

All mice were group–housed (maximum 4 mice per cage) under a 12–12 h light–dark cycle, with food and water provided ad libitum. To obtain embryos at specific time points, pregnant mice were prepared by mating females with males overnight. Noon on the day after breeding was considered embryonic day 0.5 (E0.5), and the day of birth was considered postnatal day 0 (P0).

### 4.2. Reagents

For immunostaining, the following primary antibodies were used: mouse monoclonal anti-Netrin-1 (MAB1109-100, 1:500) and goat polyclonal anti-TAG1 (AF4439; 1:200) from R&D Systems (Minneapolis, MIN, USA); rabbit polyclonal anti-Iba1 (019-19741, 1:500) from FUJIFILM Wako (Wako, Osaka, Japan). For Western blotting, the following primary antibodies were used: rabbit polyclonal anti-FAK (sc-557, 1:200) and mouse monoclonal anti-GSK3α/β (sc-7291, 1:400) from Santa Cruz Biotechnology (Santa Cruz, CA, USA); rabbit polyclonal anti-pSrc (Tyr416) (#2101, 1:1000), rabbit monoclonal anti-Src (#2123, 1:1000), rabbit polyclonal pGSK3β (Ser9) (#9336, 1:1000), rabbit monoclonal anti-pAKT (Ser473) (#4060, 1:1000), rabbit polyclonal anti-AKT (#9272, 1:1000), rabbit polyclonal anti-pERK1/2 (#9101, 1:1000), and mouse monoclonal anti-ERK1/2 (#4696, 1:1000) from Cell Signaling Technology (Danvers, MA, USA); rabbit polyclonal anti-pFAK (Tyr861) (F9176, 1:1000), rabbit polyclonal anti-pFAK (Tyr397) (ABT135, 1:1000), and mouse monoclonal anti-β-Actin (A5441; 1:3000) from Sigma Aldrich (St. Louis, MO, USA); mouse monoclonal anti-GAPDH (HC301, 1:5000) from Transgene (Beijing, China); Alexa Fluor and HRP-conjugated secondary antibody against mouse, goat, or rabbit were purchased from Sigma Aldrich. Alexa Fluor 488-phalloidin (#A12379) was obtained from Thermo Fisher Scientific (Waltham, MA, USA). IKVAV peptides, with 99% purity, were purchased from Shanghai GL Biochem Ltd. (Shanghai, China). Recombinant Human Integrin α6β1 protein (Flag & His Tag) was obtained from Sino Biological (CT013-H2508H). Recombinant Human Netrin-1 protein was purchased from Enzo Life Science (ALX-522-100-C010).

### 4.3. Cell Culture and Collection of Netrin-1 Conditioned Medium

BV2 cells were maintained in RPMI-1640 supplemented with 10% fetal bovine serum FBS and 100 units/mL of penicillin-streptomycin. HEK293T cells were cultured in Dulbecco’s modified Eagle’s medium (DMEM) supplemented with 10% FBS and 100 units/mL of penicillin-streptomycin. Netrin-1 conditioned medium was prepared as previously described [17]. Briefly, Netrin-1 plasmid was transfected into HEK293T cells using the Lipofectamine 2000 (Invitrogen) according to the manufacturer’s instructions. Twenty-four hours following transfection, the culture medium was changed into Opti-MEM without serum. Twenty-four hours later, the conditioned medium was collected, concentrated, and stored at –80 °C. The concentration of Netrin-1 in conditioned medium was quantified by Western blotting, with recombinant mouse Netrin-1 protein (1109-N1, R&D Systems) as the loading control. The concentration of Netrin-1 used in this study was 100 ng/mL.

### 4.4. In Vitro Cell Migration Assay

The in vitro cell migration assay was performed using trans-well chambers (24-well inserts, Corning 8-μm pore size, Corning). Briefly, 2 × 10^5^ cells in 200 µL of migration buffer consisting of RPMI-1640 and 0.1% BSA (bovine serum albumin) were added to the top chambers, while 600 µL of migration buffer containing Netrin-1 conditioned medium was added to the lower chambers. The cells were then cultured for 18 h in a CO_2_ incubator. After that, the cells were washed with PBS, fixed with ethanol, and stained with 0.1% Crystal Violet. The cells on the upper face of the filters were removed with cotton swabs, and the cells on the lower face of the filters were counted under a microscope.

### 4.5. Immunostaining

The brain tissues were fixed with 4% PFA/PBS overnight at 4 °C, dehydrated in 30% sucrose/PBS for 2 days, and sectioned at a thickness of 40 μm. For immunostaining, the brain slices were subjected to antigen retrieval in 0.01 M sodium citrate buffer (pH 6.0) at 98 °C for 5 min. After washing with PBS, the slices were blocked with 5% BSA in 0.2% Triton X-100/PBS for 1 h, followed by incubation with primary antibodies overnight at 4 °C. After washing in PBS supplemented with 0.1% Tween-20, the brain sections were incubated with appropriate Alexa-conjugated secondary antibodies for 1 h. To stain the extracellular Netrin-1, the slices were blocked with 5% BSA without Triton X-100.

### 4.6. Immunoblotting

Brain tissues or cultured cells were homogenized in optimized radio immunoprecipitation assay (RIPA) buffer [50 mM Tris-HCL (pH 7.4), 150 mM NaCl, 5 mM EDTA, 1% Nonidet P-40, 1% sodium deoxycholate, 0.1% SDS, 1 mM NaF, 1 mM Na3VO4, and a protease inhibitor cocktail]. The lysates were separated by SDS-PAGE and transferred to PVDF membranes. The membranes were incubated with diluted primary antibody overnight at 4 °C, followed by incubation with the appropriate HRP-conjugated secondary antibody. The blots were visualized with the ECL Prime Western Blot Detection reagent (GE Healthcare, Little Chalfont, Buckinghamshire, UK) and Tanon 5200 Automatic Chemiluminescence Image Analysis System (Tanon Science and Technology, Shanghai, China).

### 4.7. Microscale Thermophoresis (MST) Measurement

Binding affinities of Integrin α6β1 and Netrin-1 proteins were measured using MST experiments. Before the MST experiment, all protein samples were diluted in PBS containing 0.05% Tween-20 (PBST). His-tagged Integrin α6β1 was labeled with the reactive dye RED-tris-NTA using the Monolith His-tag Labeling Kit (MO-l008, NanoTemper Technologies, Munich, Germany). Then, 10 μL of serially diluted Netrin-1 proteins from 0.03 pM to 1.2 μM were loaded into sixteen PCR tubes. Next, 10 μL of 50 nM RED dye-labeled Integrin α6β1 was mixed into each reaction tube. After incubation for 10 min at room temperature, samples were filled into Monolith capillaries (MO-K022), and the binding affinity measurements were carried out using a modified manufacturer’s protocol (Monolith NT.115, NanoTemper Technologies, Munich, Germany). Experiments were performed three times with independent dilution series. The MO. Affinity Analysis software 2.2.4 was used to analyze the interaction affinity and the dissociation constant (Kd) using the Kd fit model.

### 4.8. Imaging and Quantification

The immunostained samples were observed under a Zeiss LSM 780 confocal microscope with the ZEN 2012 software (Carl Zeiss AG, Oberkochen, Germany). Only the brightness, contrast, and color balance were optimized after imaging. The numbers of Iba1^+^ cells in the cerebral cortex were counted using the ImageJ software. All counting and measurements were performed in a blinded fashion. The statistical analysis was performed using the GraphPad Prism 9 software (GraphPad Software Inc., Boston, MA, USA). The unpaired 2-tailed Student’s *t*-test following the normality distribution test was used for comparisons between two groups. One-way ANOVA followed by the LSD post hoc test was used for comparisons between three groups. The data are presented as the mean ± standard error of the mean (SEM). *p* values < 0.05 are considered statistically significant.

## Data Availability

The data that support the findings of this study are available from the corresponding author upon reasonable request.
